# Supplementary material for: FNIP1 Modulates B Cell Receptor Signaling Strength by Coordinating Metabolism During Development
Source: bioRxiv. 2026 Apr 1:2026.03.30.715168. Preprint. [Version 1] doi: 10.64898/2026.03.30.715168 (PMC13060193; doi:10.64898/2026.03.30.715168)
Supplement: Supplement 2 [file NIHPP2026.03.30.715168v1-supplement-2.pdf]

Figure S1

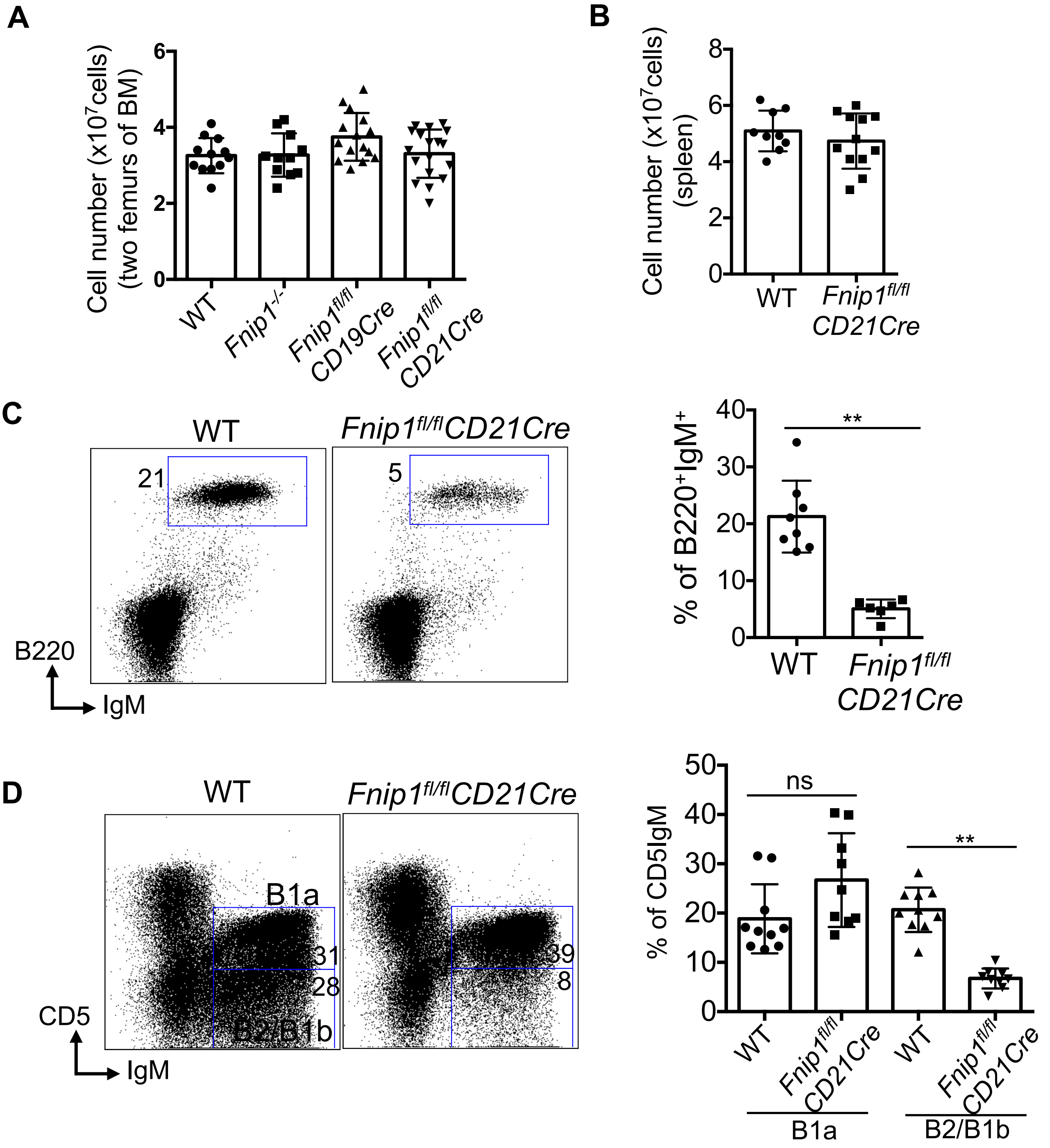

A
Cell number (x10^7cells) (two femurs of BM)
6
4
2
0
WT
Fnip1-/-
Fnip1fl/fl CD19Cre
Fnip1fl/fl CD21Cre
B
Cell number (x10^7cells) (spleen)
8
6
4
2
0
WT
Fnip1fl/fl CD21Cre
C
WT
Fnip1fl/flCD21Cre
21
5
B220
IgM
% of B220+IgM+
40
30
20
10
0
**
WT
Fnip1fl/fl CD21Cre
D
WT
Fnip1fl/flCD21Cre
B1a
31
28
B2/B1b
39
8
CD5
IgM
% of CD5IgM
50
40
30
20
10
0
ns
**
WT
Fnip1fl/fl CD21Cre
WT
Fnip1fl/fl CD21Cre
B1a
B2/B1b

Figure S2

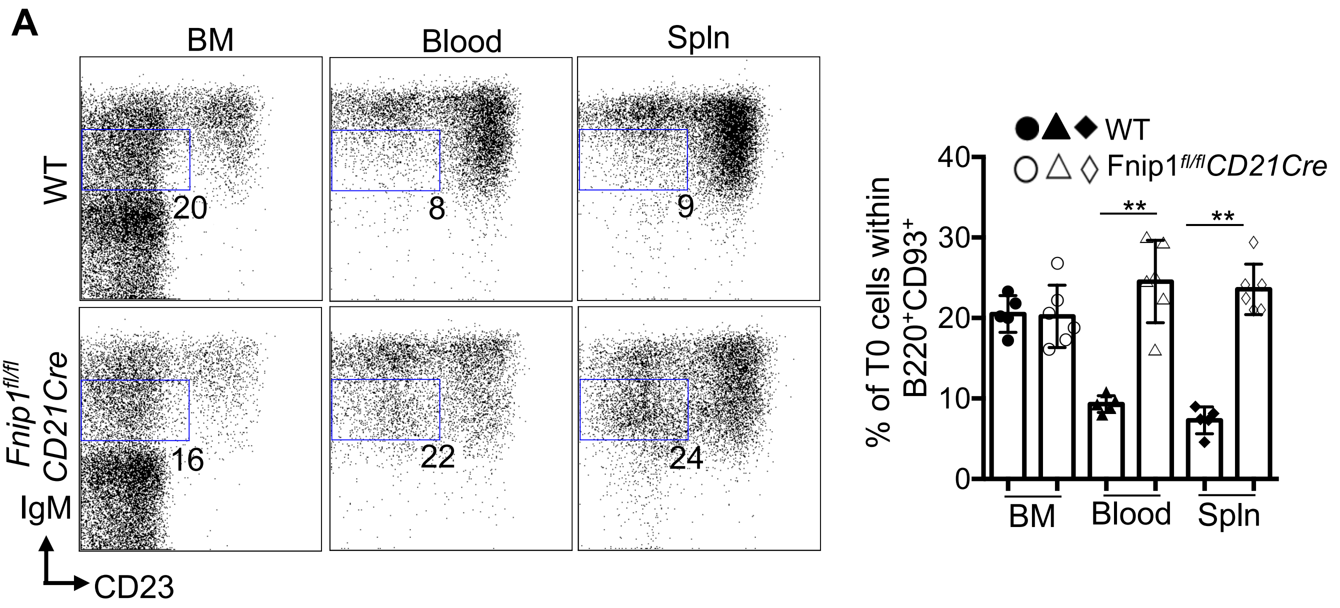
A
BM
Blood
Spln
WT
20
8
9
Fnip1fl/fl CD21Cre
16
22
24
IgM
CD23
WT
Fnip1fl/fl CD21Cre
% of T0 cells within B220+CD93+
40
30
20
10
0
**
**
BM
Blood
Spln

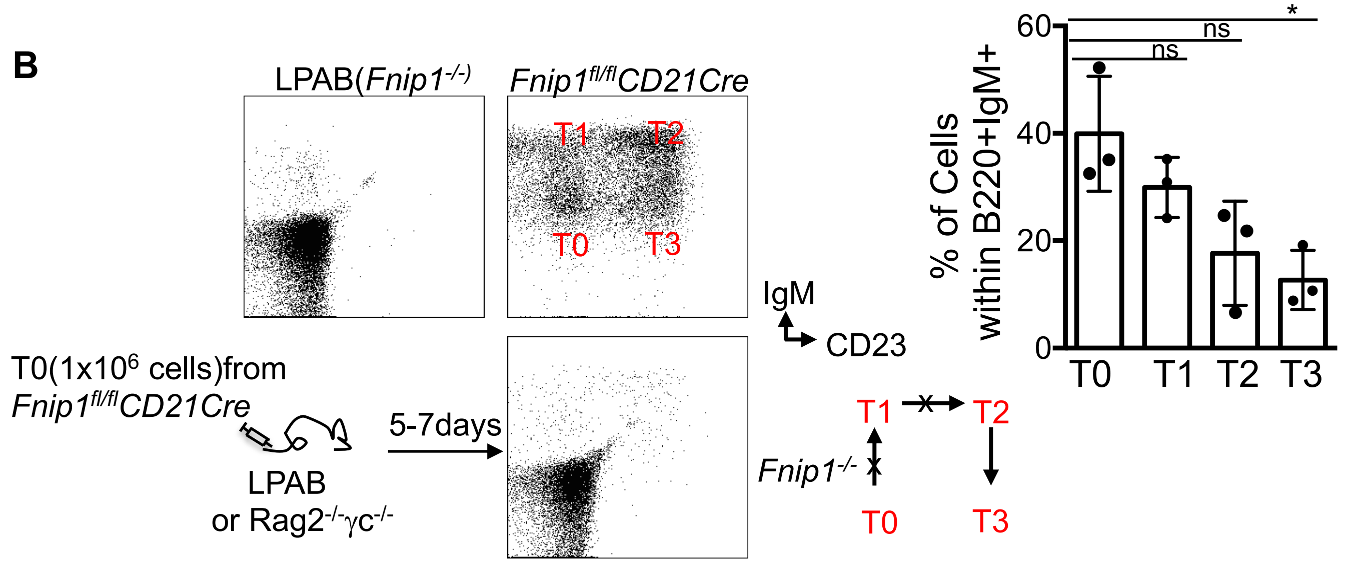
B
LPAB(Fnip1-/-)
Fnip1fl/fl CD21Cre
T1
T2
T0
T3
IgM
CD23
T0(1x10^6 cells)from
Fnip1fl/fl CD21Cre
LPAB
or Rag2-/-γc-/-
5-7days
T1
T2
Fnip1-/-
T0
T3
% of Cells within B220+IgM+
60
40
20
0
ns
ns
*
T0
T1
T2
T3

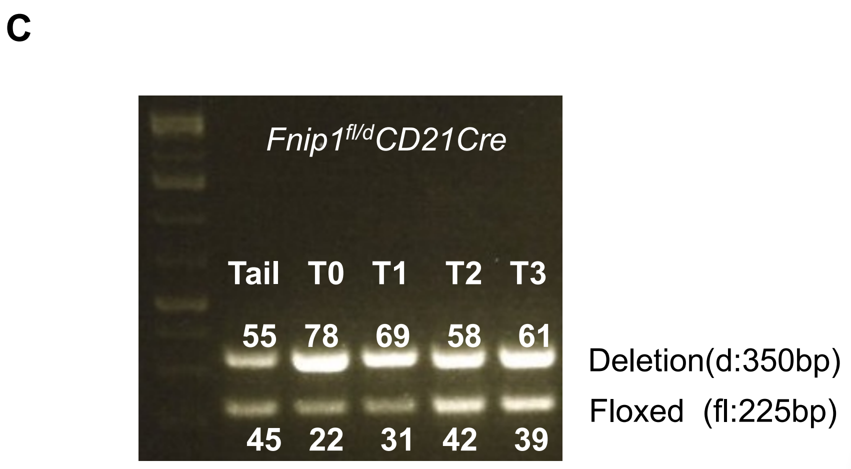
C
Fnip1fl/d CD21Cre
Tail
T0
T1
T2
T3
55
78
69
58
61
45
22
31
42
39
Deletion(d:350bp)
Floxed (fl:225bp)

Figure S3

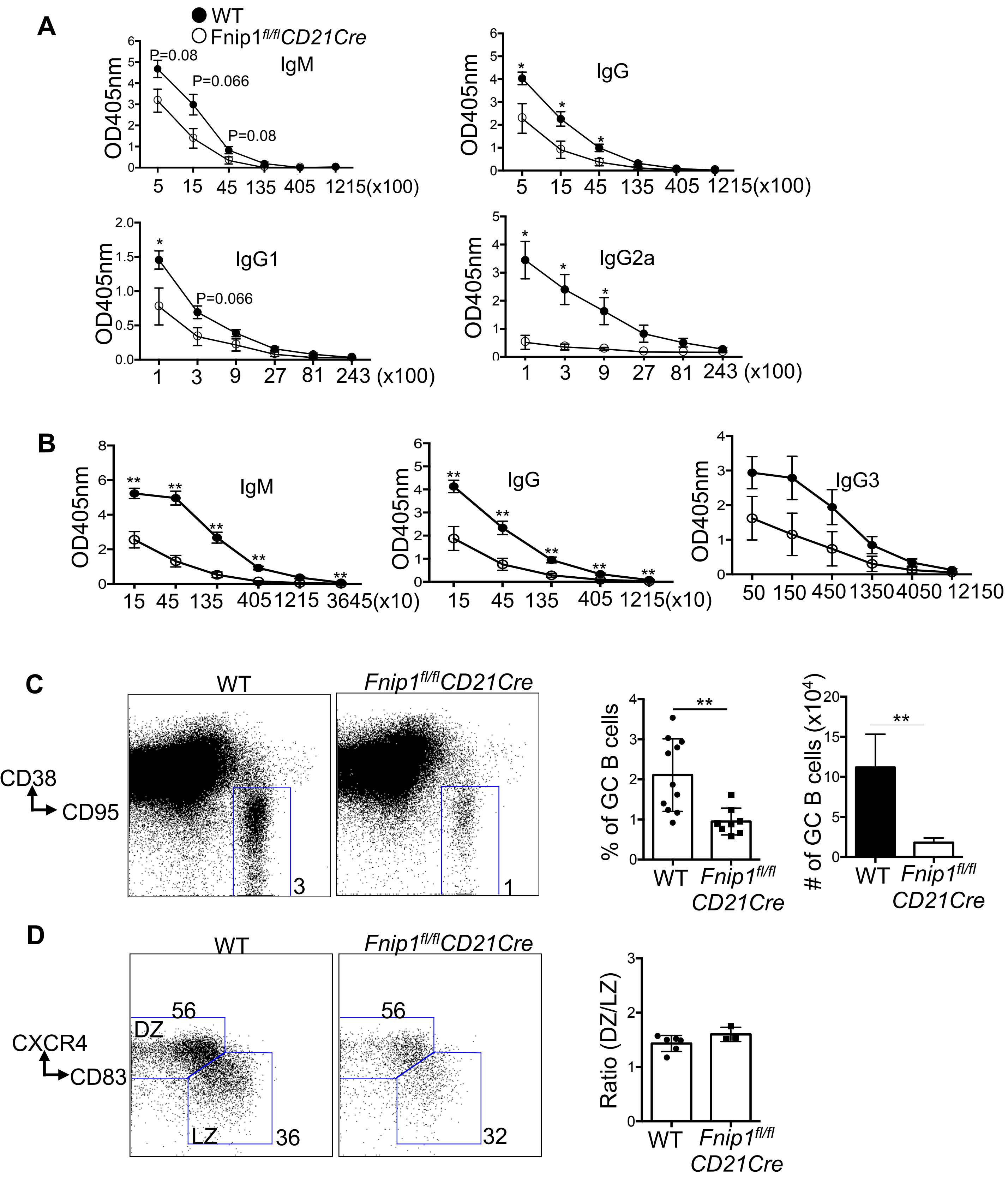
A
WT
Fnip1fl/flCD21Cre
IgM
P=0.08
P=0.066
P=0.08
OD405nm
5 15 45 135 405 1215(x100)
IgG
5 15 45 135 405 1215(x100)
IgG1
P=0.066
1 3 9 27 81 243 (x100)
IgG2a
1 3 9 27 81 243 (x100)
B
IgM
15 45 135 405 1215 3645(x10)
IgG
15 45 135 405 1215(x10)
IgG3
50 150 450 1350 4050 12150
C
WT
Fnip1fl/flCD21Cre
CD38
CD95
3
1
% of GC B cells
# of GC B cells (x10^4)
WT
Fnip1fl/fl CD21Cre
D
WT
Fnip1fl/flCD21Cre
CXCR4
CD83
56
DZ
LZ
36
56
32
Ratio (DZ/LZ)
WT
Fnip1fl/fl CD21Cre

Figure S4

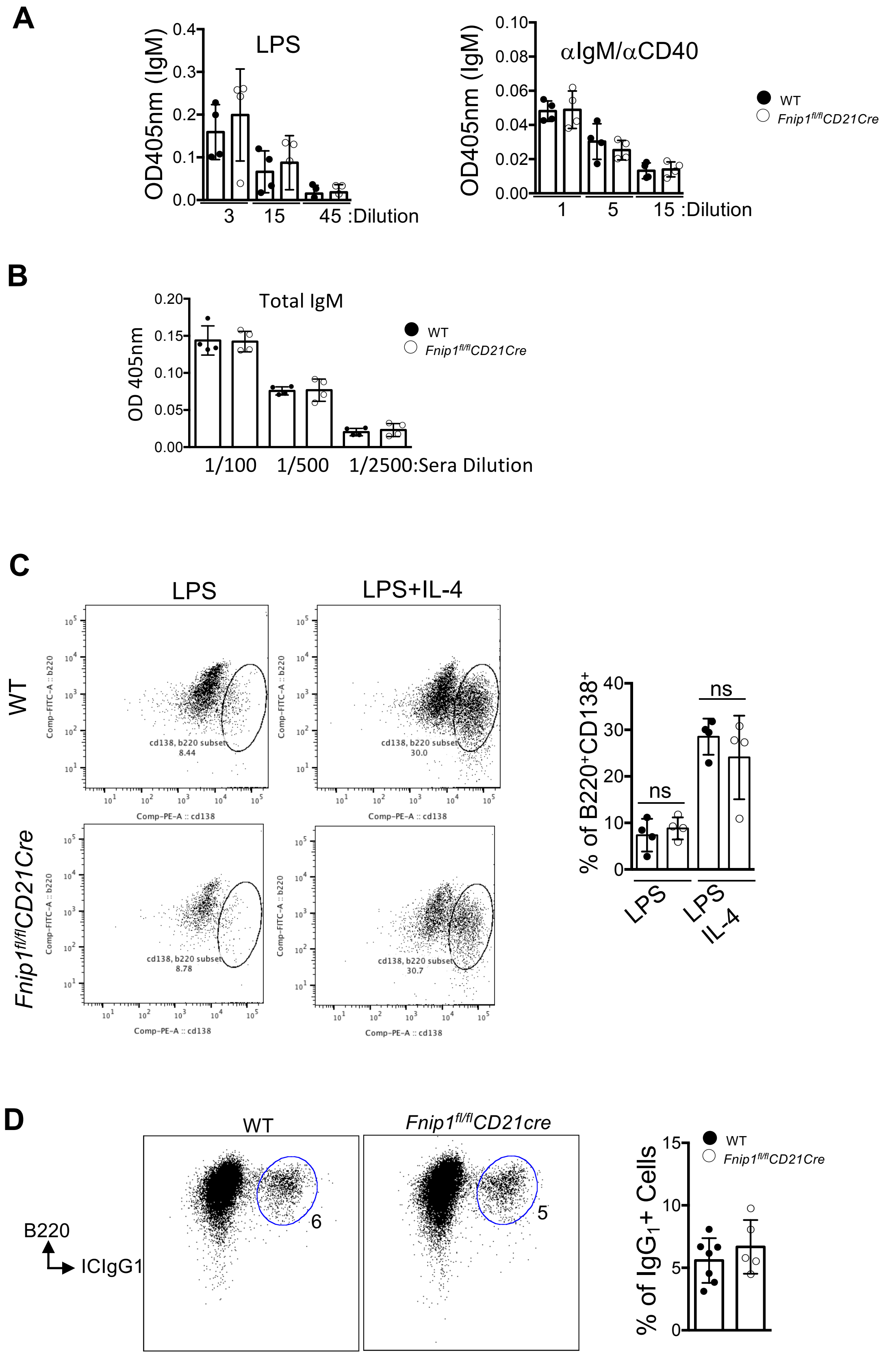
A
LPS
OD405nm (IgM)
0.0
0.1
0.2
0.3
0.4
3
15
45 :Dilution
αIgM/αCD40
0.00
0.02
0.04
0.06
0.08
0.10
1
5
15 :Dilution
WT
Fnip1fl/flCD21Cre
B
Total IgM
OD 405nm
0.00
0.05
0.10
0.15
0.20
1/100
1/500
1/2500:Sera Dilution
WT
Fnip1fl/flCD21Cre
C
LPS
LPS+IL-4
WT
Fnip1fl/flCD21Cre
Comp-FITC-A :: b220
Comp-PE-A :: cd138
cd138, b220 subset
8.44
30.0
8.78
30.7
% of B220+CD138+
0
10
20
30
40
ns
ns
LPS
LPS
IL-4
D
WT
Fnip1fl/flCD21cre
B220
ICIgG1
6
5
% of IgG1+ Cells
0
5
10
15
WT
Fnip1fl/flCD21Cre

Figure S5

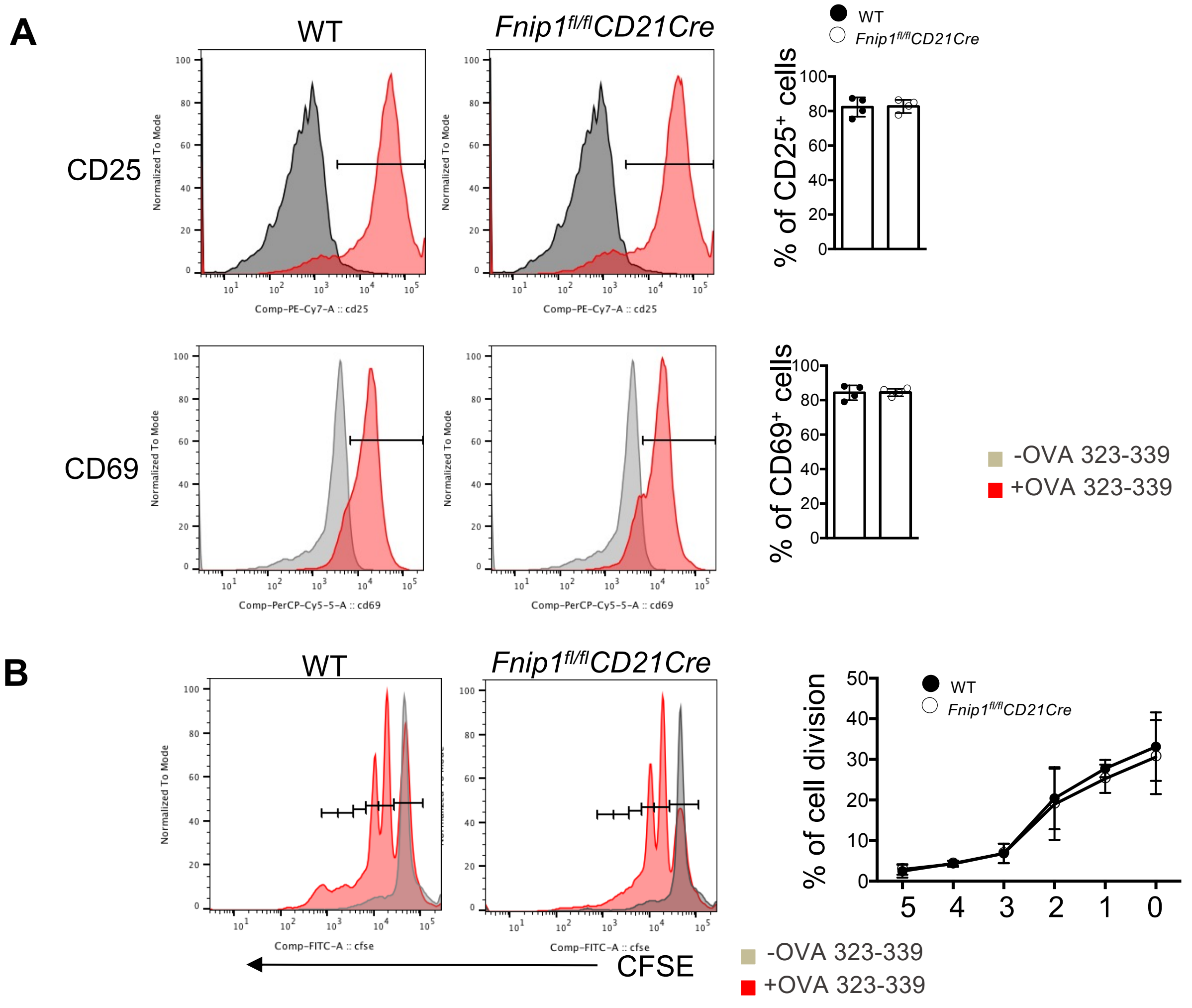

A
WT
Fnip1fl/flCD21Cre
CD25
CD69
Normalized To Mode
Comp-PE-Cy7-A :: cd25
Comp-PerCP-Cy5-5-A :: cd69
WT
Fnip1fl/flCD21Cre
% of CD25+ cells
% of CD69+ cells
-OVA 323-339
+OVA 323-339
B
WT
Fnip1fl/flCD21Cre
Comp-FITC-A :: cfse
CFSE
% of cell division
5 4 3 2 1 0
-OVA 323-339
+OVA 323-339

Figure S6

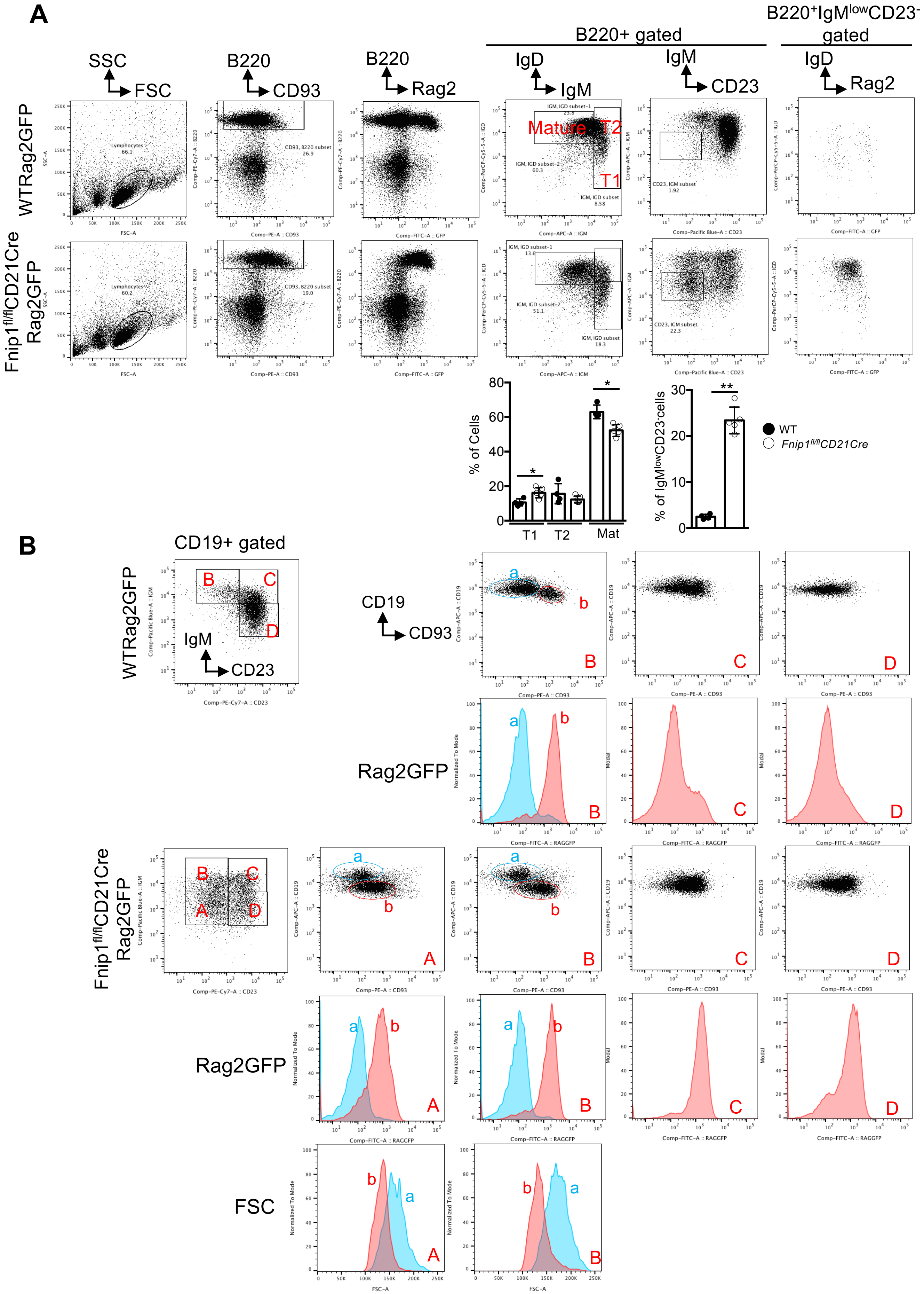
A
B220+ gated
B220+IgMlowCD23- gated
SSC
FSC
B220
CD93
B220
Rag2
IgD
IgM
IgM
CD23
IgD
Rag2
WTRag2GFP
Fnip1fl/flCD21Cre Rag2GFP
Lymphocytes 66.1
Lymphocytes 60.2
CD93, B220 subset 26.9
CD93, B220 subset 19.0
Mature
T2
T1
IGM, IGD subset-1 23.8
IGM, IGD subset-2 60.3
IGM, IGD subset 8.58
IGM, IGD subset-1 13.6
IGM, IGD subset-2 51.1
IGM, IGD subset 18.3
CD23, IGM subset 1.92
CD23, IGM subset 22.3
% of Cells
T1
T2
Mat
% of IgMlowCD23-cells
WT
Fnip1fl/flCD21Cre
B
CD19+ gated
IgM
CD23
CD19
CD93
Rag2GFP
FSC

Figure S7

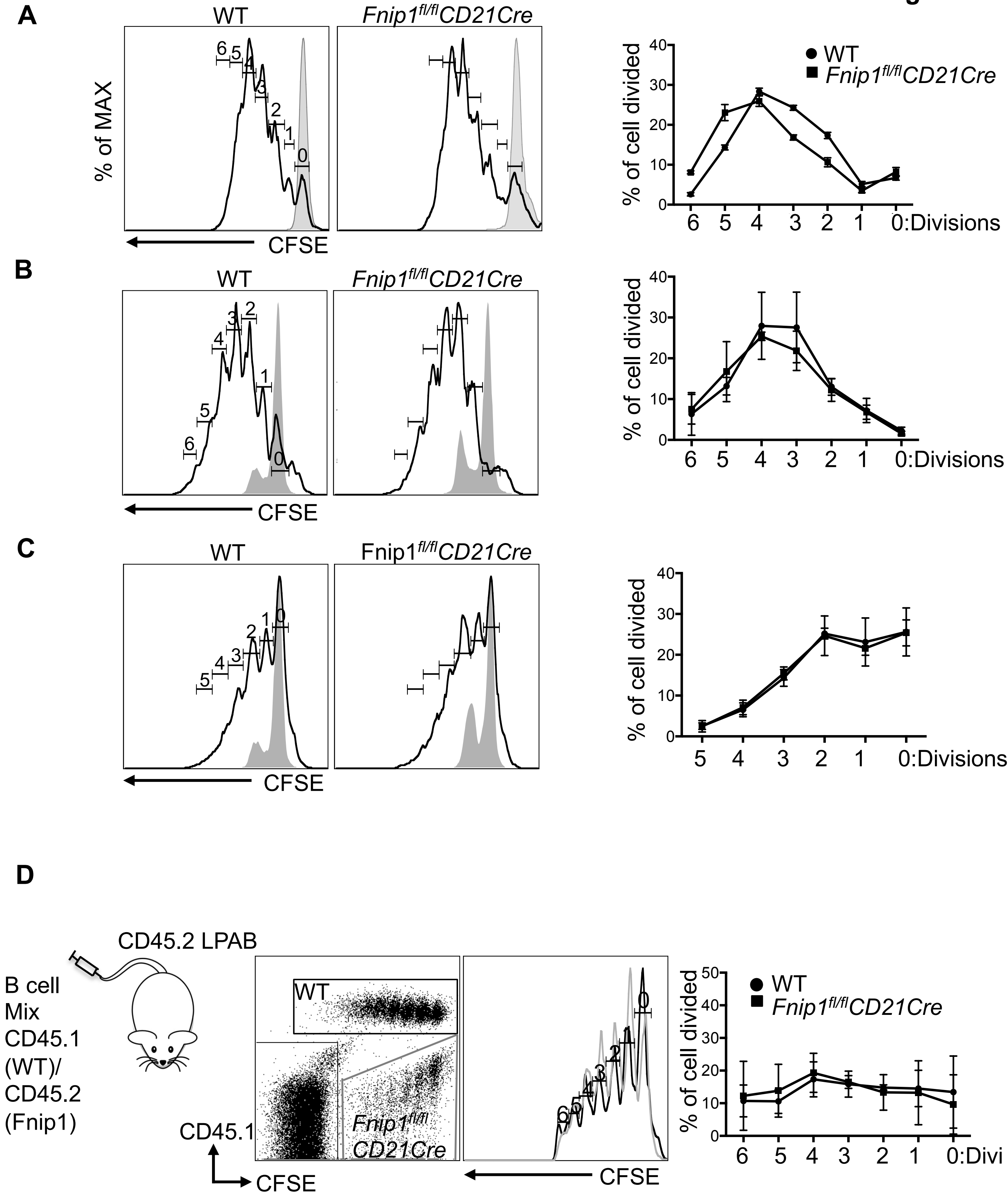
A
WT
Fnip1fl/flCD21Cre
% of MAX
CFSE
6
5
4
3
2
1
0
WT
Fnip1fl/flCD21Cre
% of cell divided
0:Divisions
B
WT
Fnip1fl/flCD21Cre
CFSE
% of cell divided
0:Divisions
C
WT
Fnip1fl/flCD21Cre
CFSE
% of cell divided
0:Divisions
D
CD45.2 LPAB
B cell
Mix
CD45.1
(WT)/
CD45.2
(Fnip1)
WT
Fnip1fl/fl
CD21Cre
CD45.1
CFSE
CFSE
WT
Fnip1fl/flCD21Cre
% of cell divided
0:Divi

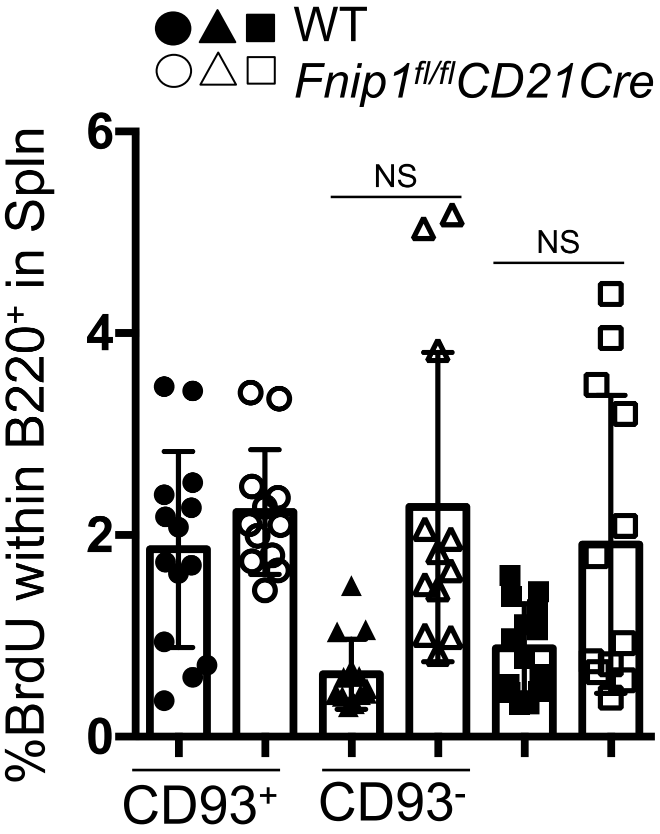
WT
Fnip1fl/flCD21Cre
%BrdU within B220+ in Spln
6
4
2
0
NS
NS
CD93+
CD93-

Figure S9

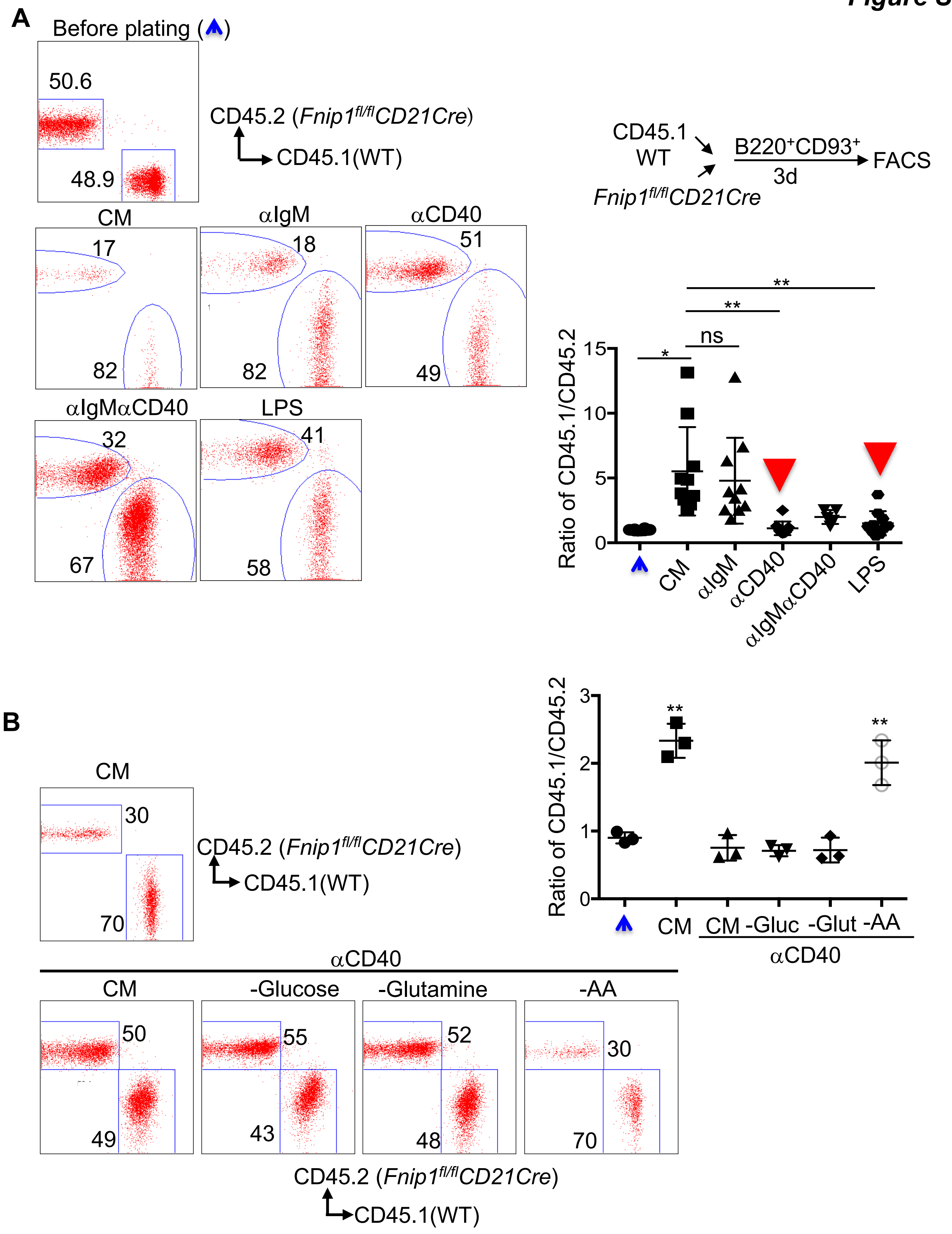
A
Before plating
50.6
48.9
CD45.2 (Fnip1fl/flCD21Cre)
CD45.1(WT)
CD45.1
WT
Fnip1fl/flCD21Cre
B220+CD93+
3d
FACS
CM
αIgM
αCD40
17
82
18
82
51
49
αIgMαCD40
LPS
32
67
41
58
**
**
ns
*
Ratio of CD45.1/CD45.2
15
10
5
0
CM
αIgM
αCD40
αIgMαCD40
LPS
B
CM
30
70
CD45.2 (Fnip1fl/flCD21Cre)
CD45.1(WT)
3
2
1
0
**
**
Ratio of CD45.1/CD45.2
CM
CM -Gluc -Glut -AA
αCD40
αCD40
CM
-Glucose
-Glutamine
-AA
50
49
55
43
52
48
30
70
CD45.2 (Fnip1fl/flCD21Cre)
CD45.1(WT)

Figure S10

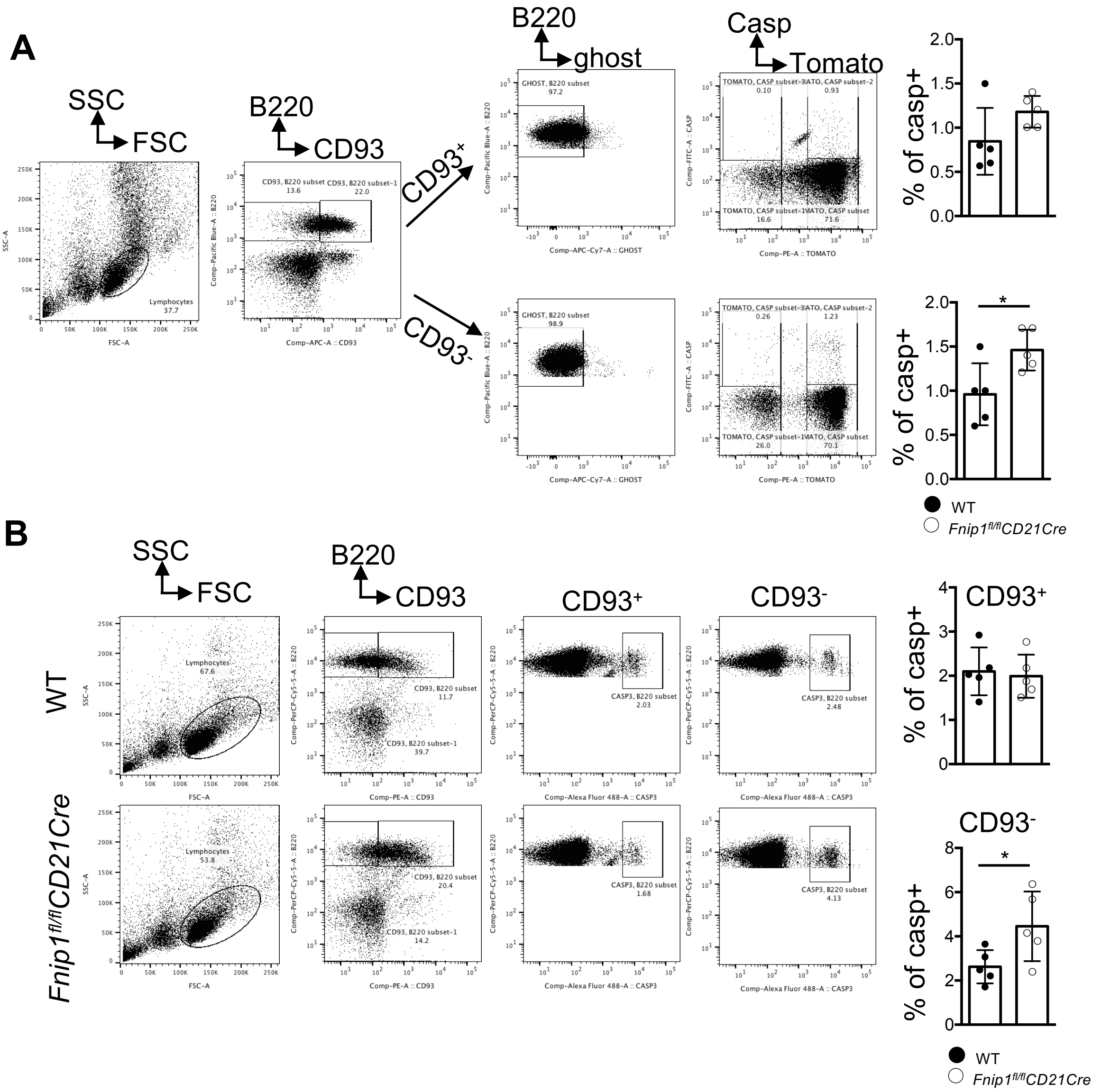
A
SSC
FSC
Lymphocytes
37.7
B220
CD93
CD93, B220 subset
13.6
CD93, B220 subset-1
22.0
CD93+
CD93-
B220
ghost
GHOST, B220 subset
97.2
GHOST, B220 subset
98.9
Casp
Tomato
% of casp+
*
WT
Fnip1 fl/fl CD21Cre
B
SSC
FSC
B220
CD93
CD93+
CD93-
WT
Fnip1 fl/fl CD21Cre
Lymphocytes
67.6
Lymphocytes
53.8
CD93, B220 subset
11.7
CD93, B220 subset-1
39.7
CD93, B220 subset
20.4
CD93, B220 subset-1
14.2
CASP3, B220 subset
2.03
CASP3, B220 subset
2.48
CASP3, B220 subset
1.68
CASP3, B220 subset
4.13
CD93+
CD93-
% of casp+
*
WT
Fnip1 fl/fl CD21Cre

Figure S11

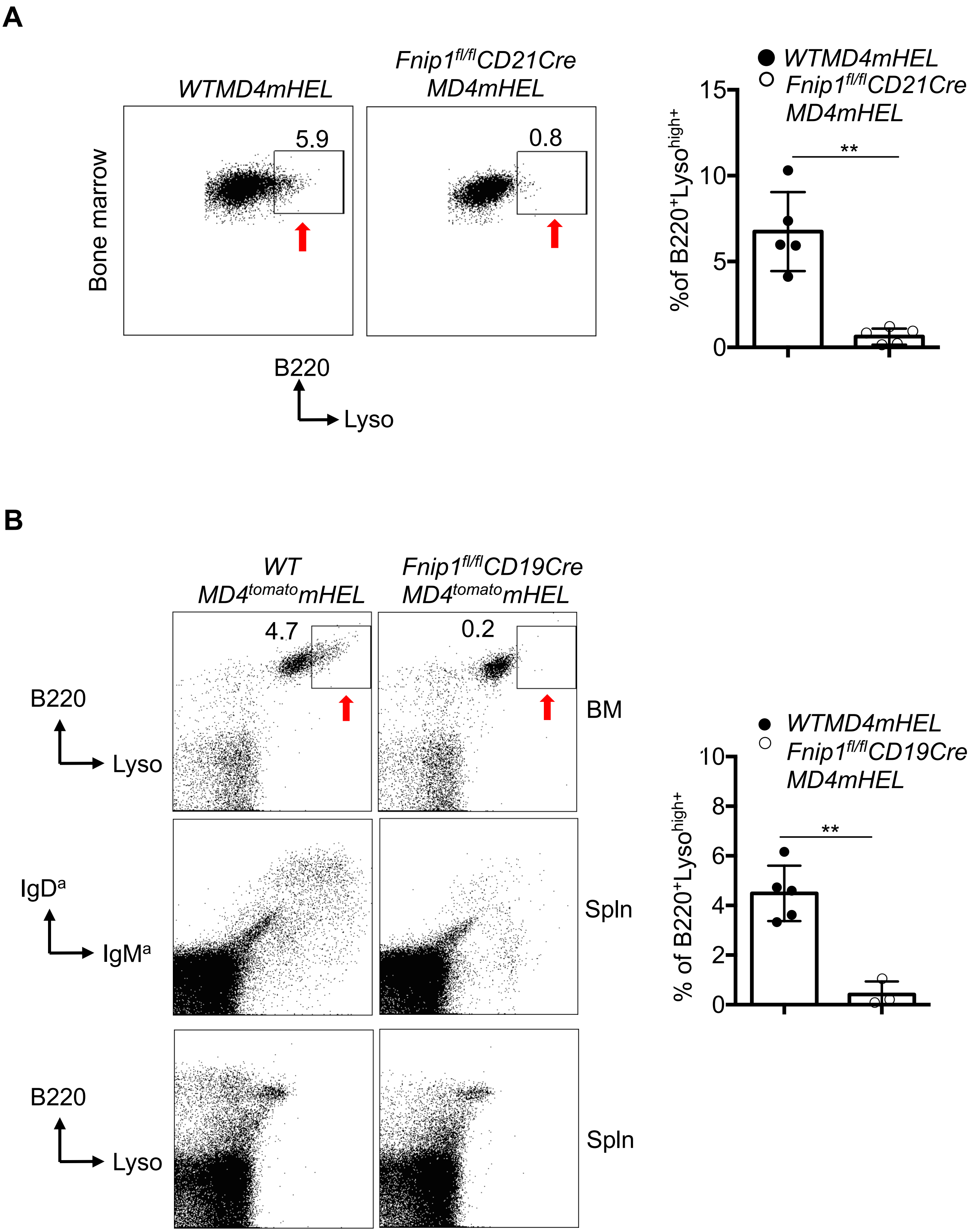
A
WTMD4mHEL
Fnip1fl/flCD21Cre
MD4mHEL
Bone marrow
5.9
0.8
B220
Lyso
WTMD4mHEL
Fnip1fl/flCD21Cre
MD4mHEL
%of B220+Lysohigh+
15
10
5
0
**
B
WT
MD4tomatomHEL
Fnip1fl/flCD19Cre
MD4tomatomHEL
4.7
0.2
B220
Lyso
BM
IgDa
IgMa
Spln
B220
Lyso
Spln
WTMD4mHEL
Fnip1fl/flCD19Cre
MD4mHEL
% of B220+Lysohigh+
10
8
6
4
2
0
**

Figure S12

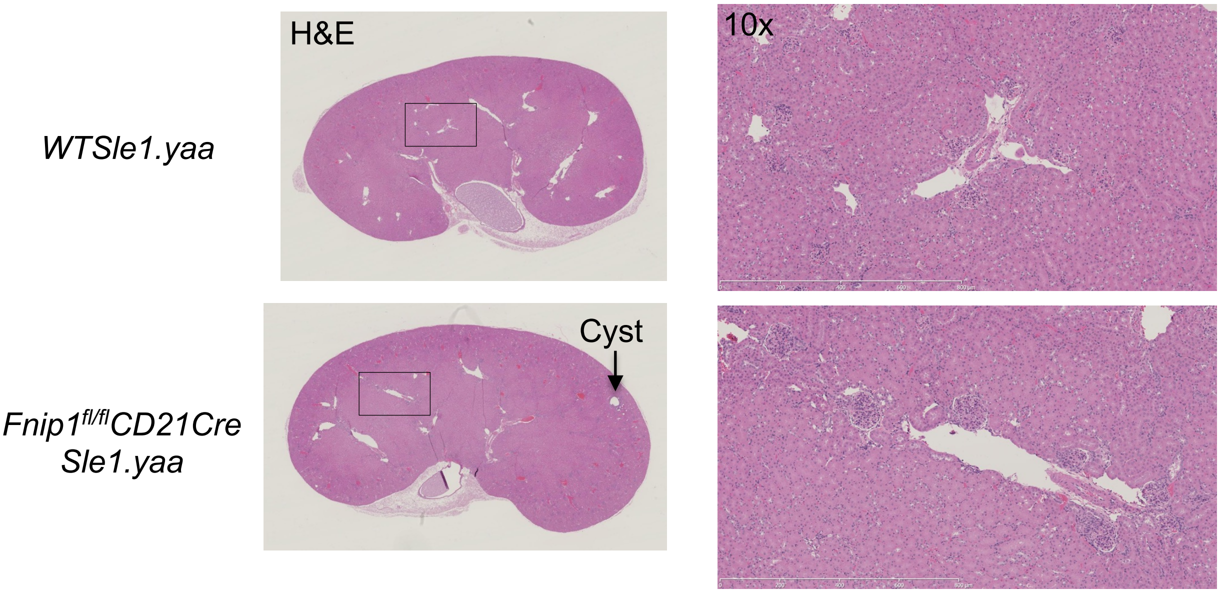

Figure S13

A

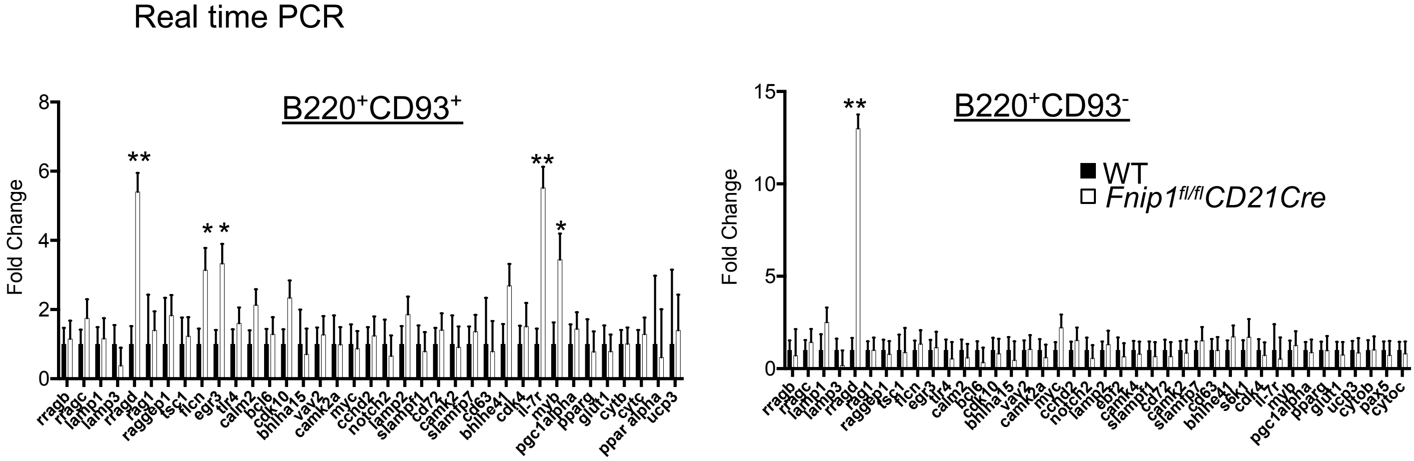

B

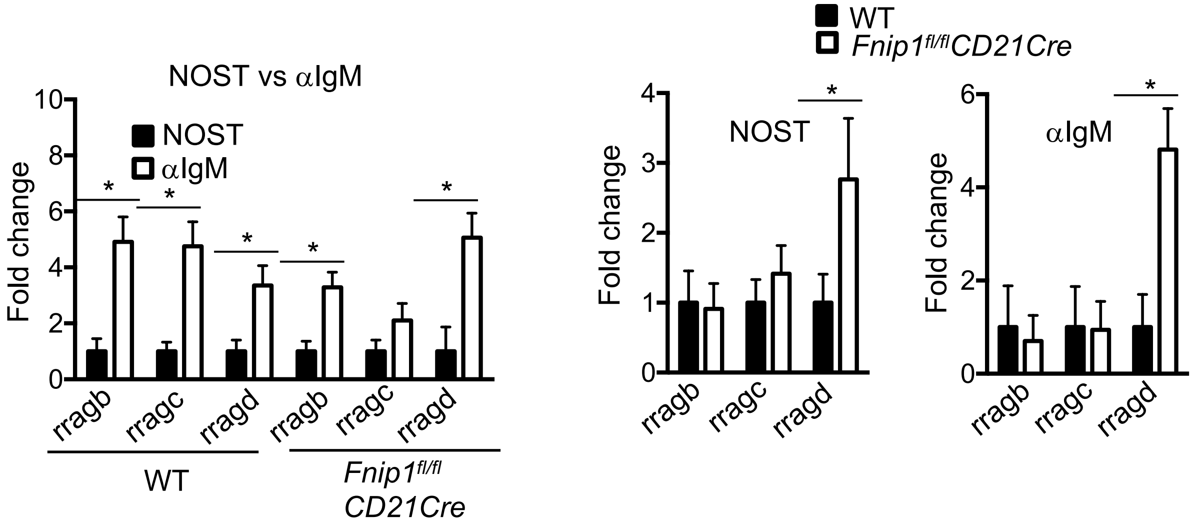

Figure S14

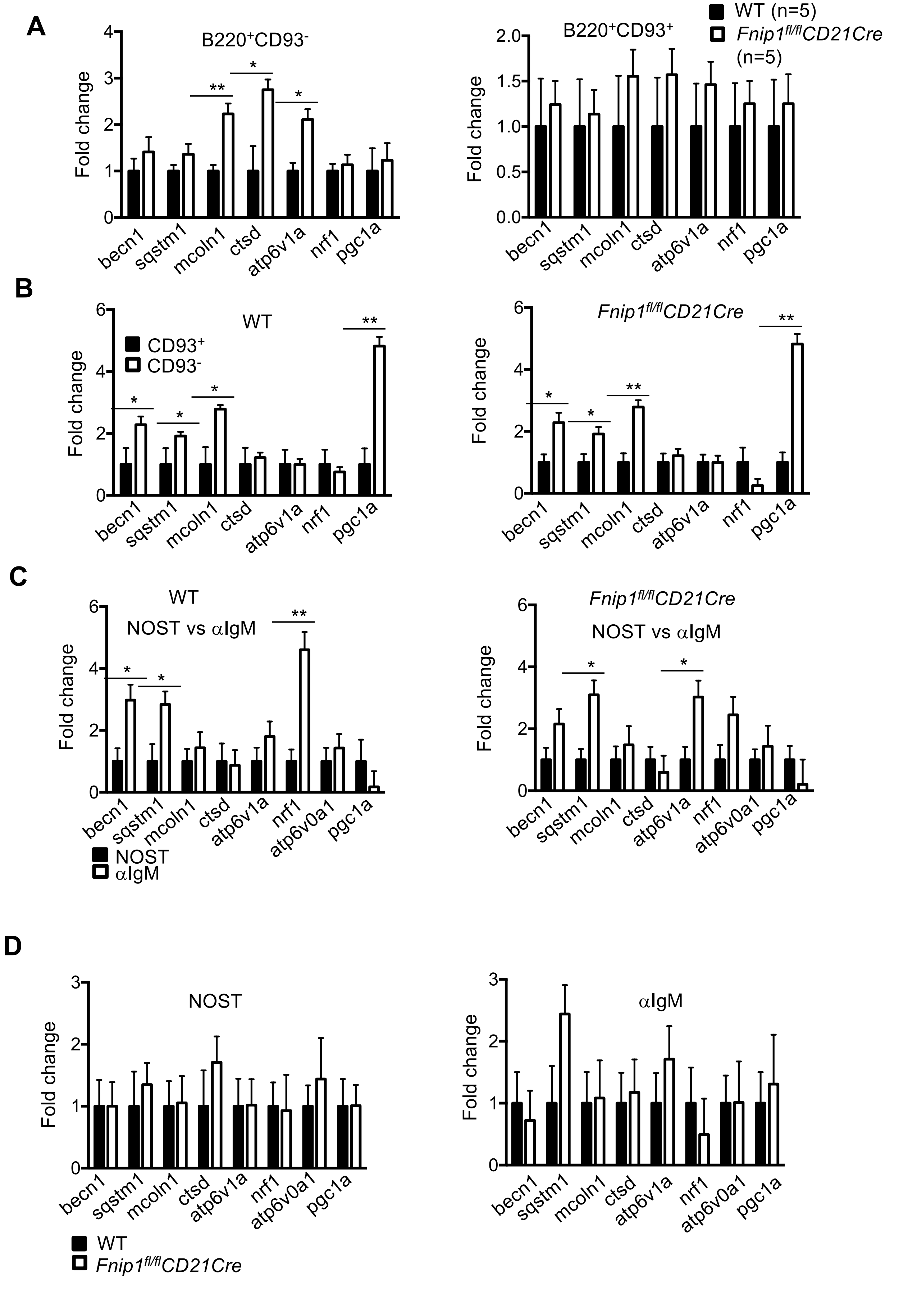
A
B220+CD93-
B220+CD93+
WT (n=5)
Fnip1fl/flCD21Cre (n=5)
Fold change
becn1
sqstm1
mcoln1
ctsd
atp6v1a
nrf1
pgc1a
B
WT
Fnip1fl/flCD21Cre
CD93+
CD93-
C
WT
NOST vs αIgM
Fnip1fl/flCD21Cre
atp6v0a1
NOST
αIgM
D
NOST
αIgM
WT
Fnip1fl/flCD21Cre

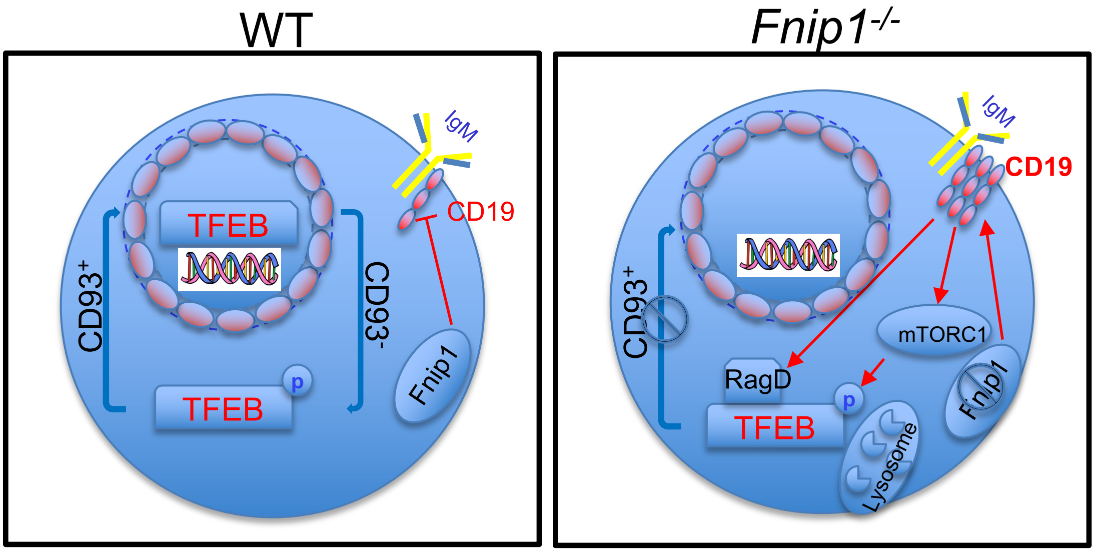

-Excessive cell growth
-CD19 upregulation
-High RagD expression
-Low lysosome contents

Figure S15. Proposed model of Fnip1 function
